# QT dispersion and cardiac involvement in patients with juvenile idiopathic arthritis

**DOI:** 10.1186/1546-0096-9-S1-P141

**Published:** 2011-09-14

**Authors:** Bulent Koca, Ozgur Kasapcopur, Suleyman Bakari, Emre Celik, Ozden Calay

## Background and aim

Juvenile idiopathic arthritis (JIA) is the commonest cause of chronic inflammatory arthritis in childhood. Cardiac involvement as pericarditis, myocarditis and valvular disease is known to occur in patients with JIA (JIA), as it does in adults with rheumatoid arthritis. There are, however, few descriptions concerning systolic and diastolic functions of the left ventricle (LV) in children with JIA. QT dispersion (QTd) is simple noninvasive arrhythmogenic marker, that can be used to assess homogeneity of cardiac repolarization, have not been studied in JIA patients before. A recent study found that rheumatoid arthritis patients had an abnormally longer QTd and corrected QT (cQTd) dispersion, markers for ventricular arrhythmogenicity. The study was to assess QTd and cQTd, their relation with systolic and diastolic function of the LV in a group of children with JIA.

## Methods

We performed electrocardiography and Doppler echocardiography on 50 patients with JIA and 70 healthy children. Maximum QT (QTmax), minimum QT (QTmin), QTd, corrected QT, maximum corrected QT (cQTmax), minimum corrected QT (cQTmin) and cQTd intervals were measured from standard 12-lead electrocardiography.

## Results

No statistically significant differences were found between the groups in QTd and cQTd. (Table 1) Among the diastolic parameters, increased late flow velocity, decreased early flow velocity and prolonged isovolumic relaxation time reflected an abnormal relaxation form of diastolic dysfunction. During 12 months of follow-up, no ventricular arrhythmias were documented in either group.

**Table 1:** Electrocardiographic measurements of the JIA patients and controls

	JIA (mean ± SD)	Controls (mean ± SD)	P value
QTmin (ms)	298.41 ± 24.52	303.38 ± 29.62	NS
QTmaks (ms)	336.82 ± 25.98	343.63 ± 28.69	NS
QTd (ms)	38.42 ± 18.45	42.57 ± 15.61	NS
cQTmin (ms)	346.91 ± 25.92	346.41 ± 29.29	NS
cQTmaks (ms)	415.40 ± 25.73	406.42 ± 27.87	NS
cQTd (ms)	67.96 ± 22.36	59.71 ± 24.02	NS

QTmin, minimum QT; QTmaks, maksimum QT; QTd, QT dispersion; cQTmin, corrected minimum QT; cQTmaks, corrected maksimum QT; cQTd, corrected

## Conclusion

In this study, QTd and cQTd was not found to be longer in JIA patients than healthy children. Larger scale, prospective, longitudinal studies are needed to assess the effect of prolonged QTd and inflammatory activity on the risk of malign ventricular arrhythmia.

